# Synthesis and evaluation of Maillard conjugates for encapsulation and controlled delivery of quercetin under simulated gastrointestinal tract conditions

**DOI:** 10.1002/fsn3.4329

**Published:** 2024-07-10

**Authors:** Angel H. Cabrera‐Ramírez, Marisol Manríquez‐Medina, Laura E. Romero‐Robles, Rocio A. Chavez‐Santoscoy

**Affiliations:** ^1^ Tecnologico de Monterrey School of Engineering and Science Monterrey Nuevo León Mexico

**Keywords:** bioactive components, drug delivery system, healthy foods, in vitro study, interaction mechanisms, Maillard conjugates, microencapsulation, quercetin

## Abstract

Encapsulation of bioactive molecules for therapeutic use is gaining great interest in the scientific community. Several encapsulation methodologies have been evaluated, sacrificing, in some cases, either encapsulation efficiency or compound integrity. Our work developed Maillard conjugates (MCs) based on the whey protein (WP)‐Maltodextrin (MD) interaction to encapsulate quercetin by freeze‐drying. The WP:MD ratio used (1:2 or 1:3) yielded the formation of MCs, demonstrated by an increased browning index and changes in the protein secondary structure. Freeze‐drying showed high encapsulation efficiency, reaching 87.65% and 84.72% in treatments loaded with 3.3 mg quercetin/g MCs. Quercetin‐loaded MCs showed spherical‐shape (4–10 μm) and a negative charge, suggesting colloidal stability. Moreover, in vitro tests demonstrated a sustained release of quercetin throughout the oral, gastric, and intestinal phases, highlighting the MCs efficacy as bioactive delivery systems. This work provides useful information to design bioactive compound delivery systems for food and pharmaceutical applications.

## Graphical abstract

Maillard conjugate (MCs) preparation induced significant changes in protein secondary structure, reducing β1 and augmenting α‐helices. Encapsulation of quercetin in MCs achieved efficiencies of up to 87.65%, thus enhancing targeted delivery. Electrical properties showed an average zeta potential of −10.37 mV, suggesting colloidal stability of MCs. MCs exhibited controlled release of quercetin throughout the oral, gastric, and intestinal phases, reaching 92.5%.

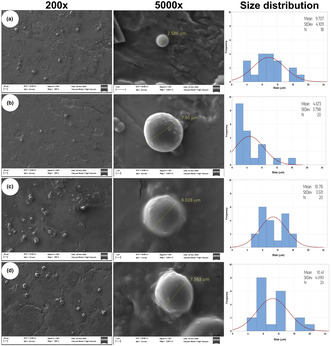

## 1 INTRODUCTION

Neurodegenerative diseases (NDDs) encompass a range of complex neurological disorders, including Alzheimer's disease, Parkinson's disease, Huntington's disease, amyotrophic lateral sclerosis, among others. The primary risk factor contributing to NDDs is aging, but human genetics and environmental factors can influence the progression of the disease (Hou et al., 2019; Maher, 2019). NDDs are the primary cause of physical and cognitive disabilities, affecting approximately 15% of the global population, a figure projected to double by 2040, largely due to the growing elderly population (Van Schependom, & D'haeseleer, 2023). One strategy to prevent NDDs is to search for natural molecules that exhibit multiple biological activities, such as vitamins, phenolics, and peptides, among others, that have the potential to affect different pathologies (Maher, 2019). Quercetin, a flavonoid commonly found in fruits and vegetables, has been documented for its diverse range of health‐supporting bioactivities, such as antioxidant, anti‐asthmatic, anti‐carcinogenic, anti‐hypertensive, and anti‐diabetic properties (Kandemir et al., 2022). Among its attributes, considerable attention has been devoted to its neuroprotective potential (Islam et al., 2021). Notably, quercetin has demonstrated neuroprotective effects against Alzheimer's disease by inhibiting amyloid‐beta plaque formation (Khan et al., 2019) and inhibiting Nrf2 ubiquitination and promoting binding of Nrf2 to DNA, thus enhancing Nrf2 transcription (Grewal et al., 2021). It has also been shown to reverse synaptic loss induced by lipopolysaccharide in the cortex and hippocampus of adult mice brains (Khan et al., 2018), and protect against rotenone‐induced neuroinflammation and alterations in mice behavior (Jain et al., 2022).

However, the potential health benefits of quercetin are limited due to its low bioavailability resulting from its low solubility in water, gastric and small intestine fluids, which makes it precipitate. Conversely, if it is glycosylated, it becomes too water‐soluble and may not be absorbed correctly. Other factors that affect bioavailability of quercetin include its high chemical instability in the gastrointestinal tract (GIT), the presence of other food components consumed with it, its botanical origin, and host‐associated factors (Kandemir et al., 2022). Since bioactive compounds are easily degraded in GIT conditions, new delivery systems—specifically, protein‐based ones—have been developed to incorporate them into functional foods. Protein‐based nanoparticles (NP) are used to deliver drugs to improve their absorption since NP can penetrate the small intestinal epithelium. Nonetheless, using only protein may lead to aggregation and precipitation at pH near their isoelectric point and high ionic strengths, so combining them with polysaccharides can result in more stable nanoparticles (Nooshkam & Varidi, 2020).

Several strategies and mechanisms have been evaluated for the encapsulation of bioactive compounds. In that sense, approaches such as the formation of liposomes have been used for quercetin encapsulation, improving its solubility and bioavailability, and demonstrating an improvement in its antioxidant activity against intracellular ROS (Rezaei‐Sadabady et al., 2016). Likewise, nanoemulsions and nanoparticles have been effective systems for quercetin delivery, targeting specific tissues or cells, and consequently showing a more efficient delivery mechanism compared to other methods (Malaj et al., 2010; Mishra & Kulkarni, 2022; YaLi et al., 2021). Polymer‐based encapsulation is another methodology employed, which has increased the bioavailability and solubility of quercetin while protecting it from degradation. Techniques such as the supercritical antisolvent process using Pluronic F127 poloxamers have been used, favoring homogeneous dispersion and increased delivery (Fraile et al., 2014; Nathiya et al., 2014). Among polymer‐based encapsulation methods, Maillard conjugates (MCs) stand out as a simple, safe, and FDA GRAS‐rated technology (Sun et al., 2022). MCs are formed through the Maillard reaction, a vast and complex group of non‐enzymatic reactions, that begin with a covalent bond between the amino groups of proteins and the carbonyl group of reducing sugars. This reaction is affected by pH, amino‐carbonyl group mass ratio, temperature, relative moisture, water activity, and time (de Oliveira et al., 2016). Choosing the right source of protein and carbohydrates to obtain MCs plays a key role in allowing the Maillard reaction to take place, ensuring food safety, and biocompatibility with the organism. Whey protein (WP) has been used for MCs production, as it is safe and biocompatible, which has been proven to be an effective strategy to improve the solubility and bioavailability of several molecules (Falsafi et al., 2022; Feng et al., 2016). As a carbohydrate source, maltodextrins (MD) are the products of the partial hydrolysis of starch and are biocompatible biopolymers that have been used to encapsulate bioactive compounds; specifically, they have been shown to enhance stability under in vitro digestion conditions and maintain stable antioxidant activity after storage (Lourenço et al., 2020). MD‐WP carriers were found to have better encapsulation efficiency, water solubility, and a smaller particle size than those with MD or WP alone (Choi et al., 2010) and compared to other carriers like gum arabic (Lekshmi et al., 2021).

Although Maillard conjugates have been extensively used in the literature for the encapsulation of bioactive compounds and essential oils, most studies perform encapsulation either by spray drying or, in other cases, by nanoemulsions (Aminikhah et al., 2023; Đorđević et al., 2015; Lourenço et al., 2020; Milea et al., 2021; Moeller et al., 2018; Wang et al., 2022). However, quercetin is a hydrophobic, thermolabile, and photosensitive compound that may be susceptible to degradation due to processing effects, which could compromise its beneficial effects. Freeze‐drying encapsulation could be a viable method for encapsulating quercetin, as it has been shown in other systems to provide superior quality and better preservation of the compound of interest than other methods such as spray drying (Moeller et al., 2018). Therefore, this work proposes the development of Maillard conjugates for the encapsulation of quercetin by freeze‐drying, evaluating encapsulation efficiency, physicochemical characteristics, release kinetics, as well as its behavior during in vitro simulated conditions of the gastrointestinal tract.

## 2 MATERIALS AND METHODS

### 2.1 Materials

For this study, it was used food grade maltodextrin (419,699, Sigma‐Aldrich), whey protein (Orgasmic Superfoods; purity of 82%), Safflower oil (B Life brand; purity of 99%), and quercetin capsules (Essential Nutrition, containing 200 mg of quercetin/300 mg of excipient) were purchased at a local supermarket.

### 2.2 Obtaining Maillard conjugates loaded with quercetin

#### 2.2.1 Experimental design

Based on preliminary analysis, a 2^2^ factorial design was employed. The source of variation considered was the ratio of whey protein to maltodextrin for the formation of Maillard conjugates (1:2 and 1:3), while the other factor was the concentration of the active ingredient (5 and 3.3 mg/g of MCP).

#### 2.2.2 Synthesis of Maillard conjugates (blank)

The Maillard conjugates (MCs) were prepared using whey protein (WP) and maltodextrin (MD) following a methodology reported by Akhtar and Dickinson (2007), with some modifications. A stock solution containing maltodextrin (MD) and whey protein (WP) with a concentration of 10% solids was prepared. This solution was agitated at 600 rpm for 24 h. Once the mother solutions were fully dissolved, mixtures were prepared according to the design: 1:2 (WP:MD) and 1:3 (WP:M). Subsequently, each mixture was agitated for 24 h, then frozen at −80°C and freeze‐dried. To form the Maillard conjugate, the freeze‐dried and ground samples were placed in a Pyrex flask, which was then sealed in a desiccator with a relative humidity of 80% (achieved using a supersaturated NaCl solution). The flask was then placed in an oven at 60°C for 4 h, and subsequently, left to cool at room temperature. These treatments were referred to as blanks (not loaded with quercetin) and will be used as wall material for quercetin encapsulation by freeze‐drying in the next stage.

#### 2.2.3 Synthesis of quercetin‐loaded MCs

To synthesize the quercetin‐loaded conjugates, a 10% solution of the previously obtained MCs was prepared and mixed with a solution of quercetin in ethanol, ensuring a concentration of 3.3 and 5 mg/g of MCs. Additionally, safflower oil (B Life brand) was added to the mixture of quercetin in ethanol as a cross‐linking agent at concentrations of 10 and 20 mg/g of MCs for the treatments containing 5 and 3.3 mg quercetin/g of MCs, respectively. The mixture was thoroughly homogenized by stirring for 30 min at 600 rpm. According to preliminary studies, emulsification was performed using a UP400St ultrasonic processor (Hielscher Ultrasonics, USA) equipped with a 3 mm probe operating at 24 Hz and 40% amplitude for 60 seconds (Chalothorn & Warisnoich, 2012). Subsequently, the mixture was frozen (−80°C) and then encapsulated by freeze‐drying. The freeze‐dried sample was ground and stored at room temperature in an airtight bag for further characterization.

Finally, the treatments were coded as “a” and “b” for those processed with a 1:2 (WP:MD) ratio with 5 and 3.3 mg/g MCs, respectively. Whereas samples with a 1:3 (WP:MD) ratio loaded with 5 and 3.3 mg/g MCs were coded as “c” and “d”.

### 2.3 Characterization

#### 2.3.1 Browning index

To determine whether the Maillard reaction occurred in the samples, the Browning index was determined in the unloaded treatments obtained in Section 2.2.2, both before and after heat treatment. For this purpose, an extract was prepared by suspending 100 mg of the sample in 1 mL of 80% ethanol (in distilled water). The mixture was agitated at 600 rpm for 30 min and then centrifuged at 5000 rpm for 5 min to recover the supernatant. Finally, the supernatant was evaluated for absorbance at 420 nm (Sail et al., 2023).

#### 2.3.2 Encapsulation efficiency (EE)

To calculate the encapsulation efficiency (EE), the total quercetin and free quercetin in the quercetin‐loaded MCs systems were determined following the methodology described by Palma et al. (2014). For this purpose, quercetin‐loaded particles were dispersed in a water:ethanol solution (70:30) with a concentration of 3 mg/mL. They were then stirred for 1 minute, placed in an ultrasonic bath for 15 min, and centrifuged at 3500 rpm for 3 min. The supernatant was filtered and collected in an HPLC vial for further analysis, being considered as the total quercetin (*T*_Q_). On the other hand, to determine the amount of unencapsulated quercetin or free quercetin (*F*_Q_), ethanol was slowly added to loaded particles until they reached a concentration of 3 mg/mL. Hand and slight agitation were performed to separate the unencapsulated quercetin from the wall without breaking the matrix. An aliquot of the supernatant was taken for HPLC analysis.

Quercetin quantification was conducted using HPLC‐DAD high‐performance liquid chromatography (1260 Infinity, Agilent Technologies, Waldbronn‐Germany) with a C18 column (Agilent Zorbax SB‐Aq; 3.0 × 150 mm, 3.5 μm) stabilized at 37°C. Acidified water (0.1% formic acid) served as phase A, and acetonitrile (0.1% formic acid) as phase B, utilizing a linear gradient from 5 to 100% phase B for 10 min. A 10 μL sample was injected, and its absorbance was monitored at 375 nm. For quantification, a standard quercetin curve was employed, with concentrations ranging from 25 to 200 ppm (*y* = 4.4748*x* + 8.8893, *R* ^2^ = 0.9943). Finally, the encapsulation efficiency (EE) was calculated using Equation 1, where TQ represents total quercetin, and FQ represents the determined free quercetin:

(1) $$\mathrm{EE}\;\left(\%\right)=\frac{T_{Q}-F_{Q}}{T_{Q}}\times 100$$

#### 2.3.3 Morphology, size, and zeta potential

The morphology of quercetin‐loaded MCs was observed using a scanning electron microscope (Zeiss EVO MA25, Germany). For this purpose, a dispersion (0.5 mg/mL in water) of MCs was prepared, and a drop was placed on the sample holder, allowing it to dry in a desiccator and then were coated with a gold layer before SEM observation. Additionally, the particle size distribution in the micrographs was evaluated using ImageJ software. On the other hand, a 0.5 mg/mL aqueous dispersion of loaded MCs was utilized for evaluating the zeta potential employing a Zetasizer (Nano ZSP, Malvern Instruments, UK).

#### 2.3.4 Infrared spectroscopy (FTIR)

The infrared spectra of the unloaded and quercetin‐loaded Maillard conjugates were obtained using a PerkinElmer IR spectrometer (SpectrumOne). The IR spectra of the freeze‐dried and ground MCs were acquired in the range of 4000 to 650 cm^−1^ with eight accumulations. The infrared spectra of whey protein, as well as the 1:2 (WP:MD) and 1:3 (WP:MD) treatments without quercetin loading (blanks), underwent a deconvolution process to examine the impact of Maillard conjugate formation on the protein secondary structure using the software Origin Pro 2015 (OriginLab, Northampton, USA).

#### 2.3.5 In vitro quercetin release

Quercetin release was evaluated following the methodology described by Ayala‐Fuentes et al. (2023). In brief, a sample of 250 mg was suspended in 100 mL of PBS (pH 7.4) and stirred at 250 rpm at 37°C for 24 h. Samples of 1 mL were taken at 0, 5, 10, 15, 30, 90, 120, 150, 180, 360, and 420 min, and then at 24 h during the agitation time. Quercetin was quantified by HPLC‐DAD, following the methodology reported in Section 2.3.2. Results were expressed as the accumulation of quercetin released in relation to the incubation time (Ayala‐Fuentes et al., 2023).

##### Mathematical model

To comprehend the mechanism of the release of encapsulated quercetin by MCs, cumulative release data obtained during the first 3 h (180 min) were fitted to various mathematical models, including Higuchi (Equation 2), Korsmeyer and Peppas (Equation 3), Lindner‐Lippold (Equation 4), and Weibull (Equation 5):

(2) $$Q_{t}=k\sqrt{t}$$

(3) $$\frac{M_{t}}{M_{\infty }}=kt^{n}$$

(4) $$\frac{M_{t}}{M_{\infty }}=kt^{n}+b$$

(5) $$\frac{M_{t}}{M_{\infty }}=1-\exp\left(-{\left(\frac{t-T_{\mathrm{lag}}}{a}\right)}^{b}\right)$$

For the above models, “*t*” represents time, “*M*_t_” is the amount of drug released at time “*t*”, and “*M*_∞_” is the total amount of drug released at the end of the process. In the case of Equation 2, “*Q*_t_” is the amount of drug released at time “*t*”, “*K*” is a proportionality constant depending on the concentration of the drug in the matrix, the diffusion coefficient of the drug in the dissolution medium, and the geometry of the matrix. Regarding Equations 3 and 4, “*k*” represents a diffusion parameter depending on the properties of the drug, the matrix, and the dissolution medium; “*n*” is an exponent indicating the release mechanism, which can be by Fick diffusion, matrix relaxation, or a coupled process; and “*b*” indicates the burst effect. In Equation 5, “*T*_lag_” is the delay or latency time representing the time required for the system to start releasing the drug, “*a*” is a parameter defining the time scale of the process, and “*b*” is a shape parameter characterizing the curvature of the release curve (Ayala‐Fuentes et al., 2023; Paarakh et al., 2019).

#### 2.3.6 In vitro simulation of the gastrointestinal tract

In vitro simulation of the gastrointestinal tract (GIT) was performed following the protocol reported by Chávez‐Santoscoy et al. (2016), with modifications of Ayala‐Fuentes et al. (2023). The oral, gastric, and intestinal phases were simulated using a water bath at 37°C with agitation at 100 rpm throughout the entire assay. During the oral phase, 200 mg of MCs were dissolved in 8 mL of α‐amylase (A‐3176, Sigma‐Aldrich) solution (1.5 mg/mL) at pH 5 in a test tube and incubated for 30 min. Aliquots (1 mL) were taken at time 0 and 30. Subsequently, the gastric phase was pH‐adjusted to 2.0 with HCl (1 M) to inactivate α‐amylase, and 2 mL of porcine pepsin solution (p‐7000, Sigma‐Aldrich) was added until a concentration of 0.05 mg/mL was reached in the total volume (8 mL). The gastric phase was evaluated for 40 min, taking 1 mL of sample at times 0, 20, and 40 min. For the intestinal phase, the pH was adjusted to 8 with NaOH (1 M), and 3 mL of pancreatin solution (p‐7545, Sigma‐Aldrich) was added to reach a concentration of 0.25 mg/mL in the total volume (8 mL). The mixture was left in incubation for 120 min, and samples (1 mL) were taken at times 0, 60, and 120 min. The enzymatic reaction was stopped by subjecting the samples to a hot bath (80°C) for 2 min and allowing them to stand for 1 h. The samples were then centrifuged (10,000×*g* at 4°C) and passed through a 0.20 mm filter (Corning, New York, NY, USA). They were stored for quantification by HPLC‐DAD (see Section 2.3.2).

#### 2.3.7 Statistical analysis

The data represent the mean ± standard deviation of three independent experiments. An analysis of variance (ANOVA) with means comparison by Tukey's test (*α* = .05%) was performed using the statistical software Minitab® 21.

## 3 RESULTS AND DISCUSSIONS

### 3.1 Blank Maillard conjugate formation

**FIGURE 1:**
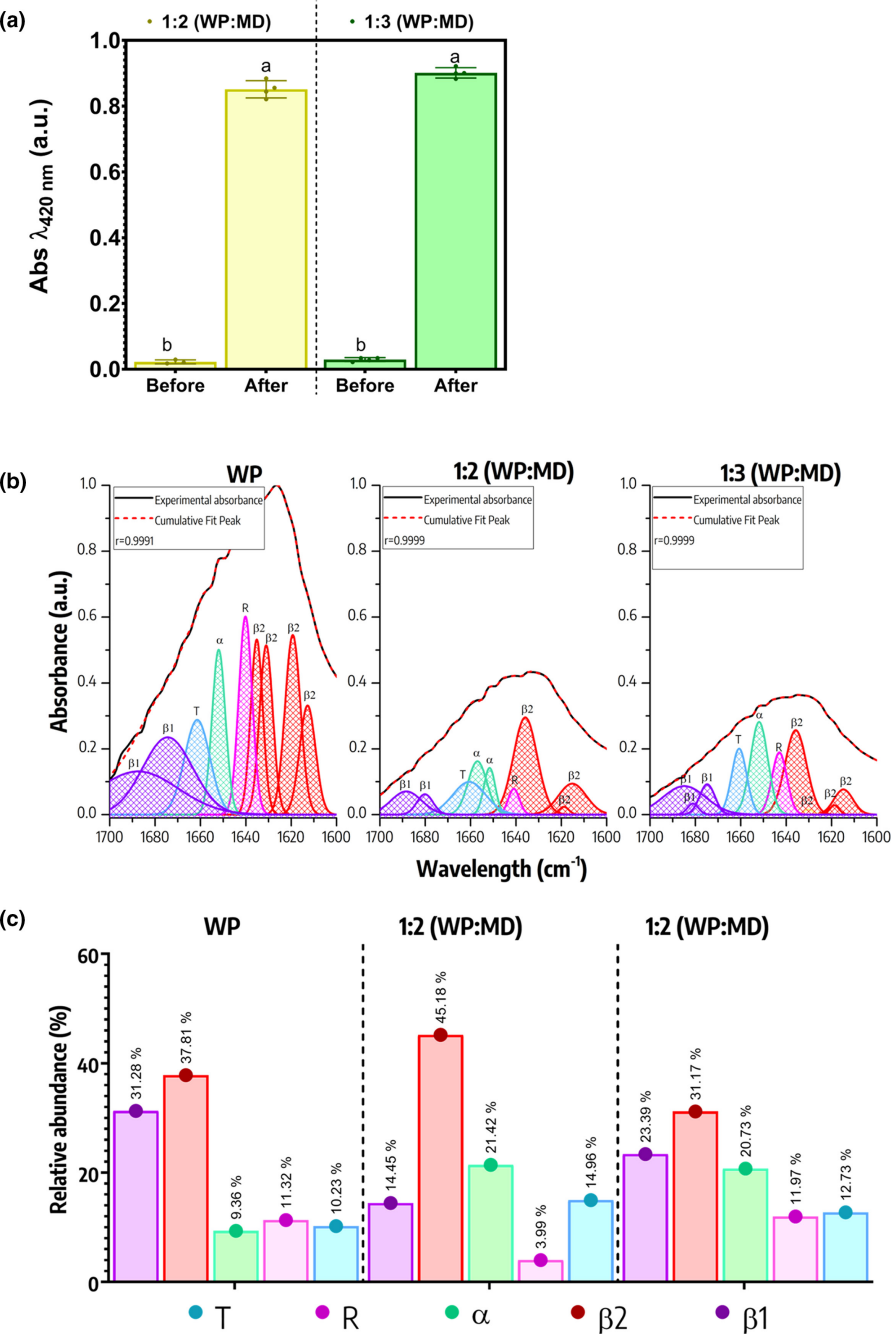
(a) Absorbance at 420 nm of extracts obtained from 1:2 (WP:MD) and 1:3 (WP:MD) treatments without loaded quercetin, both before and after heat treatment. (b) Maillard conjugate formation (blank samples) and its impact on protein secondary structure analyzed using Fourier transform infrared spectroscopy (deconvolution). (c) Relative abundance of secondary structures. MD, Maltodextrin; R, Random coil; T, ‘3‐turn’ helix; WP, Whey protein; α, α‐helix; β1, Intermolecular β‐structure; β2, Intramolecular β‐structure.

The Browning index has been utilized as a rapid indicator of the Maillard reaction (Sail et al., 2023), as illustrated in Figure 1a. In that sense, it is displayed in this figure the absorbance of the extracts obtained from the whey protein (WP) and maltodextrin (MD) treatments at 1:2 and 1:3 ratios, both before and after undergoing heat treatment to produce the Maillard conjugates (MC). Irrespective of the WP:MD ratio, it is evident that after the heat treatment, both samples exhibited an increase in absorbance at *λ*_420 nm_. This suggests that the Maillard reaction did indeed occur, resulting in the formation of colored pigments.

Infrared spectroscopy has been widely used to study the protein secondary structure, as this region is highly susceptible to changes due to processing effects (De Meutter & Goormaghtigh, 2021). Specific regions have been reported to identify the vibrations of different structures, such as the following: 1695–1670 cm^−1^ (intermolecular β‐structure), 1666–1659 cm^−1^ (‘3‐turn’ helix), 1657–1648 cm^−1^ (α‐helix), 1645–1640 cm^−1^ (random coil), and 1610–1640 cm^−1^ (intramolecular β‐structure) (Stuart, 2004). Based on the above, Figure 1b shows the analysis of the protein secondary structures using deconvolution of the infrared spectra of the WP samples, as well as the MCs obtained with the 1:2 (WP:MD) and 1:3 (WP:MD) ratio without quercetin loading (blanks). The signal intensity in the amide I region significantly decreases when going from WP to Maillard conjugates treatments, obtaining the lowest overall intensity in the 1:3 (WP:MD) treatment.

On the other hand, Figure 1c shows the relative abundance of secondary structures in the different treatments. β2 and β1 structures were the most abundant in all treatments. Particularly, β1 (intermolecular) decreased from 31% to 21%–14.45% after the formation of Maillard conjugates in both WP:MD ratios; whereas, β2 (intramolecular) vibrations remained between 37% to 45%. Therefore, the formation of Maillard conjugates can mainly affect the way in which the polypeptide chains are related to each other. In that sense, β1 vibrations represent interactions between different polypeptide chains (weak forces) and are related to protein aggregation (e.g., dipole–dipole, van der Waal, among others). While β2 (intramolecular) indicates interactions within the same polypeptide chain, which, being stronger bonds (e.g., ionic bonding, covalent bonding, among others), tend to be more resistant structures and are maintained to a greater extent during processing (Hasani et al., 2023; Stuart, 2004). Hence, explaining why after heat treatment (formation of MCs) the β1‐band (intermolecular) vibrations decreased in both treatments (1:2 and 1:3). Recently, Zhang et al. (2022) found a decrease in β‐sheets after the formation of Maillard conjugates (pea protein isolate and maltodextrin), attributed to the fact that the presence of carbohydrates prevents the interaction between protein molecules (intermolecular forces). Moreover, the authors suggest the formation of a more flexible structure with better emulsifying properties. Additionally, the α‐helices increased in both treatments up to 20%–21% compared to WP (9.36%). Such an increase could indicate the formation of more ordered structures, reinforcing the hypothesis that the Maillard reaction could be inducing the formation of cross‐bridges and linkages, thereby contributing to protein stability and organization.

### 3.2 Quercetin‐loaded MCs

#### 3.2.1 Encapsulation efficiency

**FIGURE 2:**
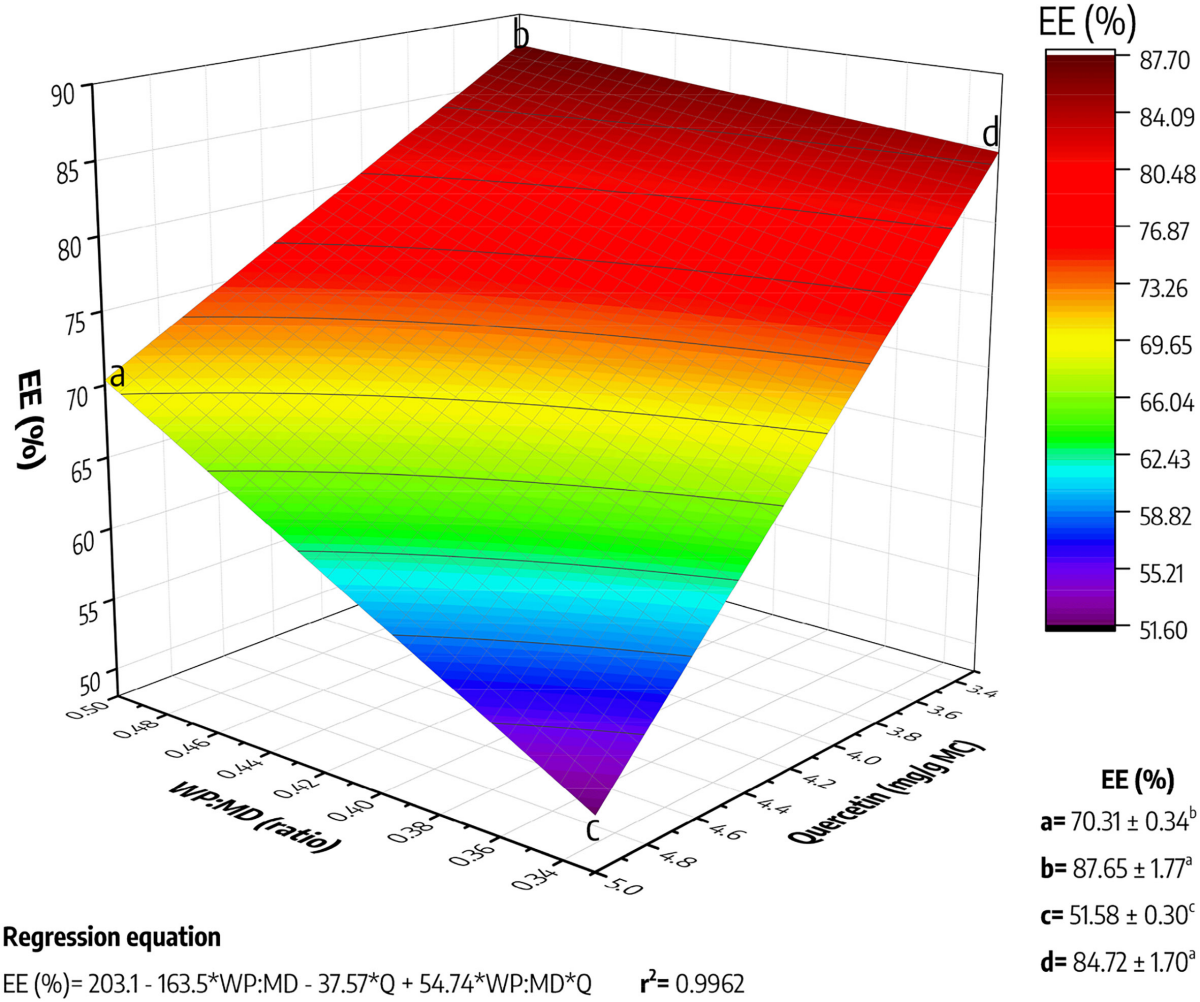
Encapsulation efficiency (EE) of quercetin in Maillard conjugates. WP:MD values represent the result of dividing the ratio; for example, 1:2 equals 0.5, or 1:3 equals 0.33. The letters “a”, “b”, “c”, and “d” represent the experimental conditions, and their achieved encapsulation efficiency is shown in the lower right corner. a: 1:2 (WP‐MD) loaded with 5 mg of quercetin/g of MCs; b: 1:2 (WP‐MD) loaded with 3.3 mg of quercetin/g of MCs; c: 1:3 (WP‐MD) loaded with 5 mg of quercetin/g of MCs; b: 1:3 (WP‐MD) loaded with 3.3 mg of quercetin/g of MCs; MD, maltodextrin; WP, whey protein.

The encapsulation efficiency (EE) of quercetin in Maillard conjugates is presented in Figure 2. It is evident that both the WP:MD ratio and quercetin concentration significantly influence the encapsulation efficiency. Concerning the WP:MD ratio, a tendency is observed where the 1:2 ratio (value 0.5 in WP:MD axis) tends to exhibit higher EE values compared to those obtained with the 1:3 ratio (value 0.33 in WP:MD axis) at the same drug concentration. Specifically, EE values of 70.31% (a) and 87.65% (b) were achieved when the MCs were loaded with 5 and 3.3 mg/g of quercetin at a 1:2 ratio (WP:MD). Conversely, the treatments processed with a 1:3 ratio resulted in an EE of 51.58% (c) and 84.72% (d) for 5 and 3.3 mg/g quercetin, respectively.

This difference could stem from the ability of whey protein and maltodextrin to interact and form Maillard complexes during thermal processing, as shown in Figure 1. A 1:2 ratio may provide an optimal amount of reactive groups in the protein and maltodextrin, leading to a higher production of cross‐links. This, in turn, results in a more balanced reaction favoring a more efficient matrix for quercetin retention. On the other hand, a 1:3 ratio, having an excess of one component (maltodextrin), could negatively impact complex formation, limiting the availability of reactive sites and thereby reducing the drug encapsulation capacity.

On the other hand, it was shown that drug concentration is a crucial factor in encapsulation efficiency. In that sense, it was evident that a lower concentration (3.3 mg/g of MCs) resulted in higher encapsulation efficiency in both WP:MD ratios. This could be partly explained by a saturation effect, in which a high concentration of quercetin may lead to increased competition for the available reactive sites, resulting in reduced encapsulation efficiency. Meanwhile, a low drug concentration could face a higher probability of interacting with the reactive groups, favoring the formation of interactions with the Maillard conjugates, and increasing their EE.

In the case of MCs loaded with 3.3 mg/g quercetin, they exhibited an EE higher than 80%, surpassing other similar studies. In this context, Gallegos‐Granados (2021) reported percentages of 71.1% using inulin as an encapsulating agent, while Shah et al. (2012) reported an encapsulation efficiency of eugenol lower than 50% using Maillard conjugates obtained from whey protein (WP) and maltodextrin (MD). However, it should be noted that both studies employed spray drying for drug encapsulation. Therefore, in our study, by utilizing freeze‐drying as a method of encapsulation, we may be avoiding thermal degradation of the drug. Due to their high encapsulation efficiency, the encapsulates obtained show great potential as functional ingredients, especially those loaded with 3.3 mg of quercetin/g of MCs.

#### 3.2.2 Physicochemical characterization of MCs

**FIGURE 3:**
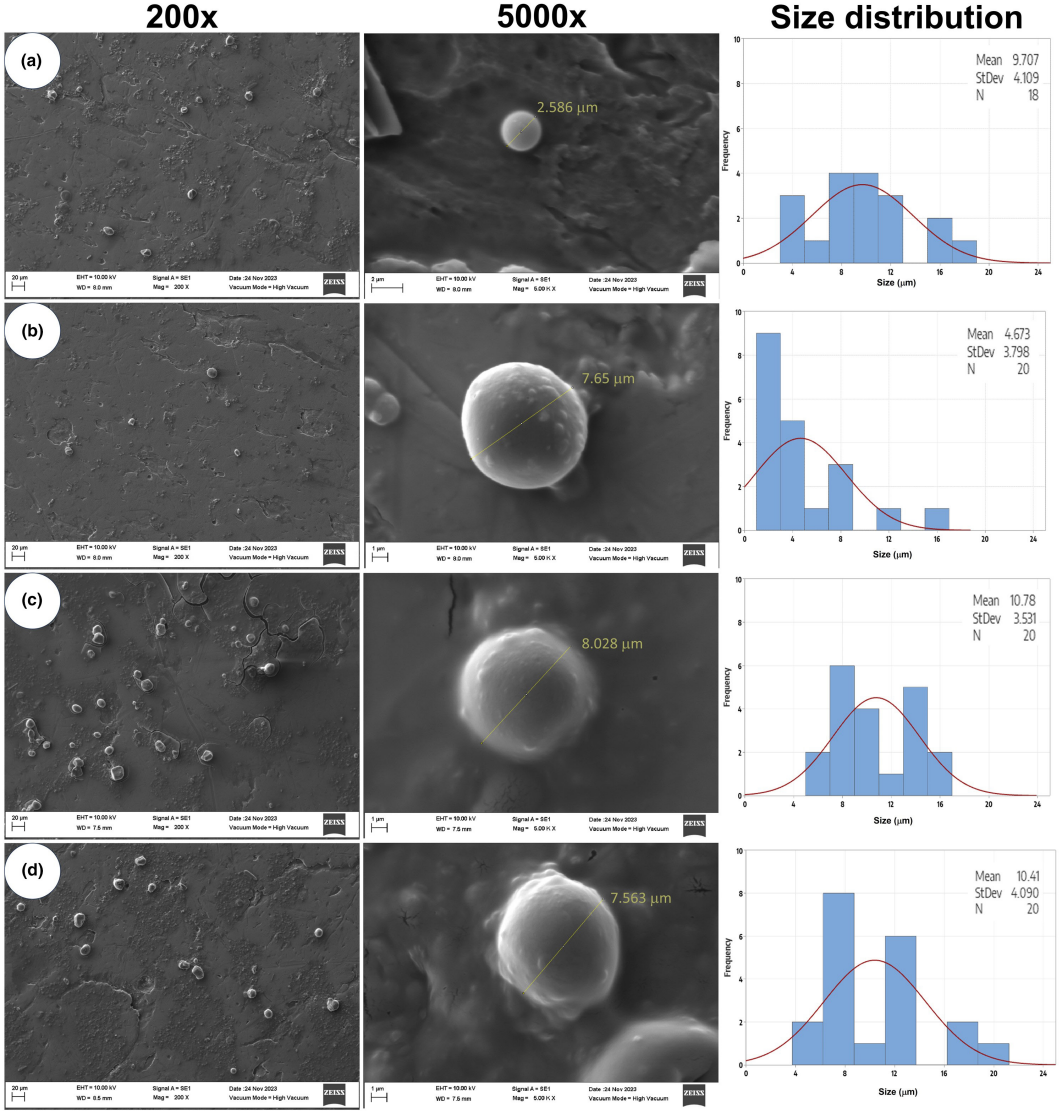
Morphology and size of MCs loaded with quercetin. (a) 1:2 (WP‐MD) loaded with 5 mg of quercetin/g of MCs; (b) 1:2 (WP‐MD) loaded with 3.3 mg of quercetin/g of MCs; (c) 1:3 (WP‐MD) loaded with 5 mg of quercetin/g of MCs; (d) 1:3 (WP‐MD) loaded with 3.3 mg of quercetin/g of MCs; MD, maltodextrin; WP, whey protein.

Figure 3 presents the morphology of quercetin‐loaded Maillard conjugates observed at 200 and 5000x magnifications. The morphology was similar for all four systems, regardless of the protein‐carbohydrate ratio used for the formation of the MCs and the amount of quercetin loaded. In this regard, the MCs presented a hemispherical morphology with the presence of some surface roughness. Although the morphology was mostly spherical, some oval shapes were also observed. Some reports have indicated that the morphology of Maillard conjugates is typically irregular and dependent on the characteristics of the wall materials (carbohydrate‐protein), the method of obtaining, and the method of encapsulation (Nooshkam et al., 2020). The morphology of quercetin encapsulated using whey protein‐inulin as a wall material was reported by Ayala‐Fuentes et al. (2023), which is similar to that found in our study. It is important to note that the size of their system was at the nanometer scale since the encapsulation was done through spray drying. On the other hand, the evaluation of particle size distribution was conducted (Figure 3), revealing that the MCs obtained fall within the range of microparticles. In this context, a trend was observed towards smaller sizes in the systems obtained with a 1:2 ratio (WP:MD), ranging from 4.67 to 9.7 μm. Meanwhile, the MCs obtained with a ratio of 1:3 (WP:MD) exhibited a size around 10 μm. Additionally, it should be noted that in both cases, a considerable variability in particle size was observed, with a standard deviation of 3 to 4 μm.

**FIGURE 4:**
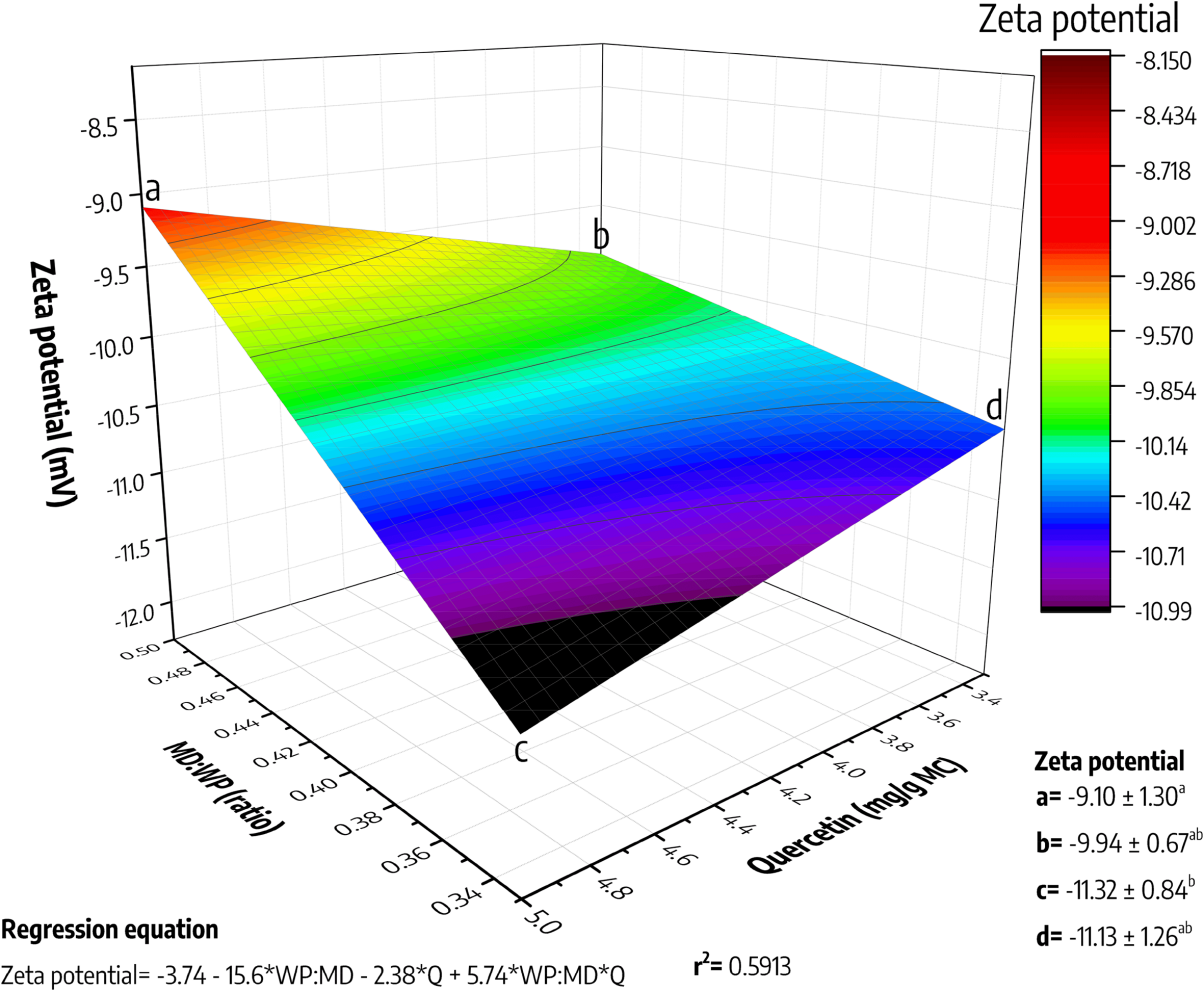
Zeta potential of Maillard conjugates loaded with quercetin. WP:MD values represent the result of dividing the ratio; for example, 1:2 equals 0.5, or 1:3 equals 0.33. The letters “a”, “b”, “c”, and “d” represent the experimental conditions, and their achieved encapsulation efficiency is shown in the lower right corner. a: 1:2 (WP‐MD) loaded with 5 mg of quercetin/g of MCs; b: 1:2 (WP‐MD) loaded with 3.3 mg of quercetin/g of MCs; c: 1:3 (WP‐MD) loaded with 5 mg of quercetin/g of MCs; b: 1:3 (WP‐MD) loaded with 3.3 mg of quercetin/g of MCs; MD, maltodextrin; WP, whey protein.

To evaluate the stability and interaction of the MCs in suspension, the evaluation of the zeta potential was performed (Figure 4). The WP:MD ratio demonstrated a significant effect on the zeta potential, with a tendency to exhibit values from −9.1 to −9.94 mV when using a 1:2 ratio (WP:MD). In contrast, when using a 1:3 ratio (WP:MD), the charge of the MCs was found to be between −11.13 and −11.32 mV, regardless of the quercetin concentration used. The zeta potential, a measure of the surface charge of the particles, as is known, provides insight into the electrostatic stability of the system (Bhattacharjee, 2016). Therefore, the 1:3 system favors a higher negative charge, potentially increasing colloidal stability. In that sense, Zhang et al. (2022) report that positively charged amino acids such as lysine are conjugated to the negative charge of maltodextrin, resulting in the formation of conjugates with a higher negative charge. This explains the observed increase in charge negativity after increasing the proportion of maltodextrin in the MCs obtained using the 1:3 ratio (WP:MD).

The zeta potential (ZP) has been widely used in pharmacology to assess the colloidal stability of different systems, generating ranges to classify their stability: highly unstable (±0–10 mV), relatively stable (±10–20 mV), moderately stable (±20–30 mV), and highly stable (> ± 30 mV) (Agrawal & Patel, 2011). As such, the data obtained would indicate that systems obtained with a 1:2 ratio are highly unstable, while those obtained with a 1:3 ratio would be considered relatively stable. Nevertheless, it should be clarified that this ZP parameter is not definitive, and its interpretation is more complex, as it does not consider van der Waals forces of attraction (Bhattacharjee, 2016). In this sense, other colloidal systems, such as colloidal silica (An et al., 2014), and water–oil emulsions (Almeida et al., 2015), have been shown to be very stable despite their low ZP values.

**FIGURE 5:**
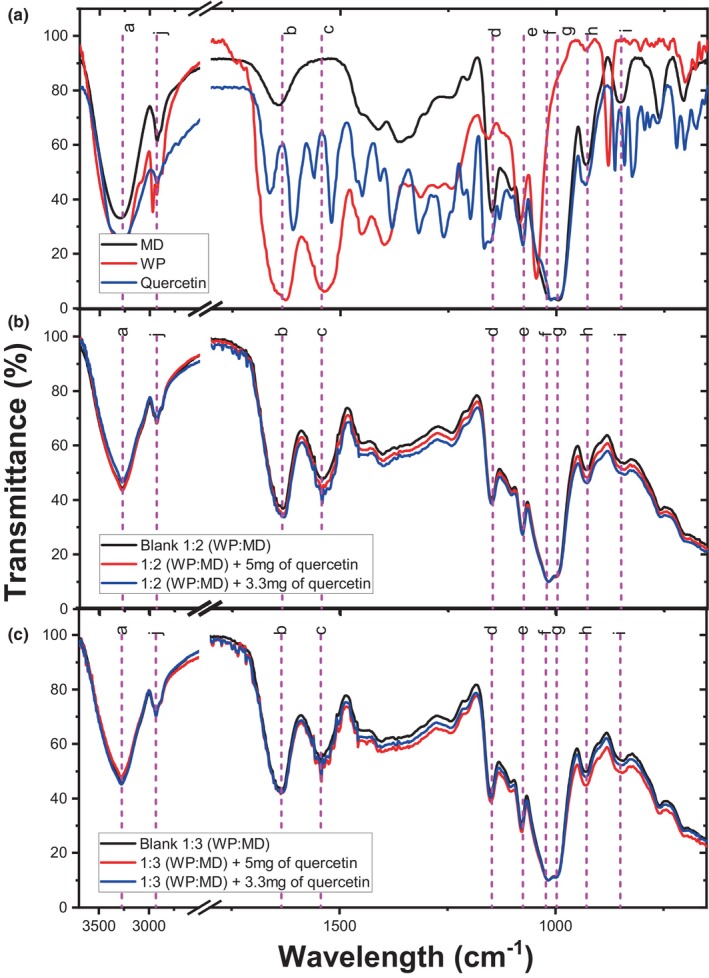
Infrared spectra of raw materials (a), and Maillard conjugates (MCs) loaded with quercetin at different WP:MD ratios. (b) MCs produced with a 1:2 ratio (WP:MD) loaded with 5 and 3.3 mg quercetin/g of MCs; (c) MCs produced with a 1:3 ratio (WP:MD) loaded with 5 and 3.3 mg quercetin/g of MCs; Blank: Represents the Maillard conjugate obtained with the same WP:MD ratio (1:2 or 1:3) without quercetin loading. The letters represent arbitrary coding to identify the main vibrations associated with the infrared spectrum of the samples.

On the other hand, Figure 5 displays the infrared spectra of pure components (WP, MD, and quercetin) and both the unloaded and quercetin‐loaded Maillard conjugates. The IR spectra remained similar to the blank, regardless of the WP:MD ratio or the addition of quercetin (MCs without quercetin loading). Bands associated with the major component, maltodextrin, were observed. Symmetrical vibrations of the C‐O groups and skeletal C‐C vibrations were appreciated at 997 cm^−1^ [g]. Deformation vibrations of the C‐O‐H groups were observed at 1022 cm^−1^ [f]. In the zone at 1075 [e] and 1147 cm^−1^ [d], deformation and antisymmetric vibrations of the C‐OH groups were identified, respectively. Symmetric vibrations of the CH_2_‐groups were found at 2980 cm^−1^ [j].

For characteristic protein vibrations (Maqsoudlou et al., 2020), vibrations of N‐H, C‐N, and C=O groups corresponding to amide bonds were observed in the region of 850 cm^−1^ [i]. Bending and symmetric stretching vibrations of the NH_3_ and CO_3_‐ groups were found in the region from 1000 to 1050 cm^−1^, respectively. Some other authors have found that in this region, vibrations of rings present in free amino acids can be present. Moreover, there was evident presence of an intense signal in the amide region (1500 to 1700 cm^−1^), where the vibration observed at 1543 cm^−1^ [c] was associated with the vibrations of the N‐H and C‐N groups related to Amide II. Furthermore, at 1635 cm^−1^ [b], the vibrations of the C=O groups were located, characteristic of Amide I. Finally, at 3271 cm^−1^ [a], vibrations of the bonds belonging to the ‐OH and hydrogen groups present in the N‐H, C‐H, and C‐OH groups were found, both in protein and maltodextrin.

After the incorporation of quercetin, a decrease in transmittance is observed in the amide II [c] and amide I [d] bands, as well as the “*h*” and “*i*” bands, suggesting a possible interaction with quercetin. This may indicate that an interaction or bond formation is taking place between quercetin and the N‐H groups belonging to the protein, and the C‐OH groups of the maltodextrin. The Maillard reaction occurs through the covalent bonding of the reducing group (‐CHO) of carbohydrates with the amino groups of the protein. This reaction is influenced by characteristics such as pH, temperature, moisture, mass ratio (amino‐carbonyl groups), and time (de Oliveira et al., 2016). Thus, the vibrational changes observed above could be indicative that the Maillard reaction indeed took place, and part of the groups reacted to allow the entrapment of quercetin. This is favored in the case of the 1:2 ratio (WP:MD) when using 3.3 mg quercetin/g of MCs. In contrast, in the case of 1:3 (WP:MD), the opposite phenomenon is observed. The above is consistent with previous data (Figures 2 and 3), where it is observed that characteristics such as encapsulation efficiency and size of Maillard conjugates depend on the WP:MD ratio as well as the concentration of loaded quercetin.

#### 3.2.3 In vitro quercetin release

**FIGURE 6:**
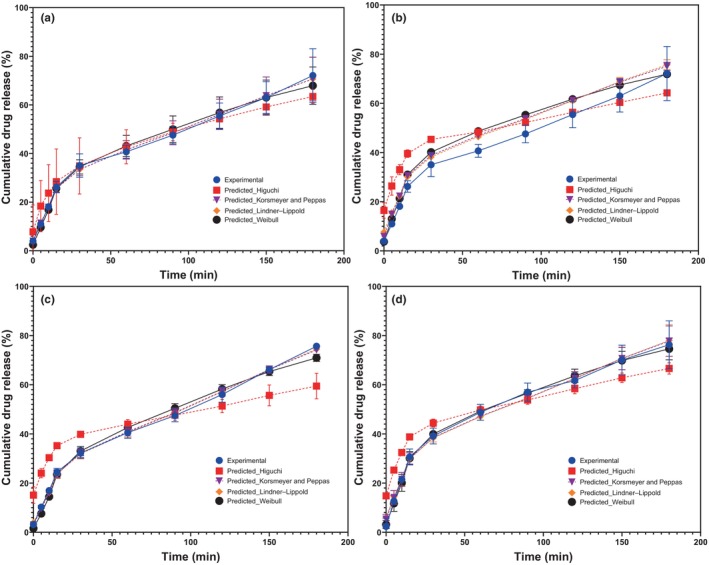
Behavior of cumulative drug release (%) from experimental data and predictions derived from different mathematical models. (a) 1:2 (WP‐MD) loaded with 5 mg of quercetin/g of MCs. (b) 1:2 (WP‐MD) loaded with 3.3 mg of quercetin/g of MCs. (c) 1:3 (WP‐MD) loaded with 5 mg of quercetin/g of MCs. (d) 1:3 (WP‐MD) loaded with 3.3 mg of quercetin/g of MCs; WP: Whey protein; MD: Maltodextrin.

The quercetin release behavior in the different treatments is depicted in Figure 6, along with the values predicted by the various mathematical models evaluated, whose parameters and determination coefficients (*r* ^2^) are presented in Table 1. The quercetin release behavior (Figure 4) exhibited a similar trend in the four systems, displaying a sigmoidal release with rapid release in the initial stages of the kinetics, followed by a slow or sustained release over time. Concerning the first stage, systems differed in the time taken to reach a release of 40% of the drug load. In this context, 1:2 (WP:MD) systems loaded with 5 (Figure 4a) and 3.3 (Figure 4b) mg quercetin/g MCs achieved 40% release in the initial 60 and 15 min, respectively. Meanwhile, the 1:3 (WP:MD) systems loaded with 5 and 3.3 mg quercetin/g of MCs reached 40% release at 30 and 15 min, respectively.

**TABLE 1:** Parameters for drug release behavior obtained from fitting to mathematical models.

Sample	Mathematical model							
	Higuchi		Korsmeyer and Peppas			Lindner–Lippold		Weibull
a	*K*_h_	6.270 ± 1.97	*K*	0.906 ± 0.13	*K*_s_	0.874 ± 0.18	*b*	1.397 ± 0.15
	*r* ^2^	.974	*n*	1.049 ± 0.07	*n*	1.056 ± 0.08	*r* ^2^	.9921
			*r* ^2^	.9972	*b*	0.209 ± 0.29		
					*r* ^2^	.9972		
b	*K*_h_	7.313 ± 0.03	*K*	1.606 ± 0.01	*K*_s_	1.002 ± 0.20	*b*	1.0275 ± 0.01
	*r* ^2^	.971	*n*	0.931 ± 0.01	*n*	1.030 ± 0.05	*r* ^2^	.9871
			*r* ^2^	.9979	b	2.382 ± 0.111		
					*r* ^2^	.9989		
c	*K*_h_	6.784 ± 0.68	*K*	0.608 ± 0.10	*K*_s_	0.449 ± 0.23	*b*	1.586 ± 0.04
	*r* ^2^	.963	*n*	1.178 ± 0.05	*n*	1.239 ± 0.12	*r* ^2^	.9891
			*r* ^2^	.9979	b	2.628 ± 0.01		
					*r* ^2^	.9983		
d	*K*_h_	7.485 ± 0.50	*K*	1.492 ± 0.91	*K*_s_	1.282 ± 0.93	*b*	1.394 ± 0.25
	*r* ^2^	.976	*n*	0.987 ± 0.16	*n*	1.166 ± 0.21	*r* ^2^	.9891
			*r* ^2^	.9881	b	2.034 ± 0.02		
					*r* ^2^	.9882		

*Note*: a: 1:2 (WP‐MD) loaded with 5 mg of quercetin/g of MCs; b: 1:2 (WP‐MD) loaded with 3.3 mg of quercetin/g of MCs; c: 1:3 (WP‐MD) loaded with 5 mg of quercetin/g of MCs; b: 1:3 (WP‐MD) loaded with 3.3 mg of quercetin/g of MCs; MD, maltodextrin; WP, whey protein.

On the other hand, parameters of the fit of the data to the different mathematical models are presented in Table 1. These models have been widely used in pharmacological studies to comprehend the release mechanism of drugs of interest (Paarakh et al., 2019). A trend indicating higher values in the constants is observed when a quercetin concentration of 5 mg/g of MCs is used. Concerning Higuchi's model, the *K*_h_ constant serves as a measure indicating the rate of drug release from a porous matrix per unit time (Ahmed et al., 2019). Therefore, the Higuchi constant (*K*_h_) suggests that quercetin release follows a kinetics that propagates proportionally to the square root of time. Treatments loaded with 3.3 mg quercetin/g MCs appear to have a more accelerated release rate compared to treatments loaded with 5 mg.

The Korsmeyer and Peppas model is commonly employed to elucidate the release behavior of drugs retained in polymeric matrices. Specifically, the parameter “*n*” is an exponent indicating the release mechanism, which can be Fick diffusion, relaxation of the matrix, or a coupled process (Talevi & Ruiz, 2021). According to the results obtained in Table 1, treatments loaded with 5 mg of quercetin per gram presented values of “*n*” > 1, suggesting a Super Case Type II transport mechanism. Such behavior has been associated with chain relaxation being the driving mechanism for release (Talevi & Ruiz, 2021). On the other hand, treatments loaded with 3.3 mg quercetin showed values of “*n*” < 1, associated with an Anomalous (non‐Fickian transport) release mechanism (Ayala‐fuentes et al., 2022). This could suggest that drug interaction with the delivery system material, system heterogeneity, or the presence of physical or chemical barriers could be affecting drug diffusion. For the Lindner‐Lippold model, the “b” parameter presented positive values, indicating an initial burst effect on drug release in the four systems evaluated (Ayala‐fuentes et al., 2022), thus explaining the behavior previously observed in the time required to reach a release of 40% (Figure 4). Finally, using the Weibull model, the “b” parameter showed values greater than 1, indicating fractal or disordered diffusion. Similarly, this fractal diffusion could be interpreted as the matrix of the encapsulant being porous or irregular, potentially affecting the release pattern.

#### 3.2.4 In vitro gastrointestinal digestion

**FIGURE 7:**
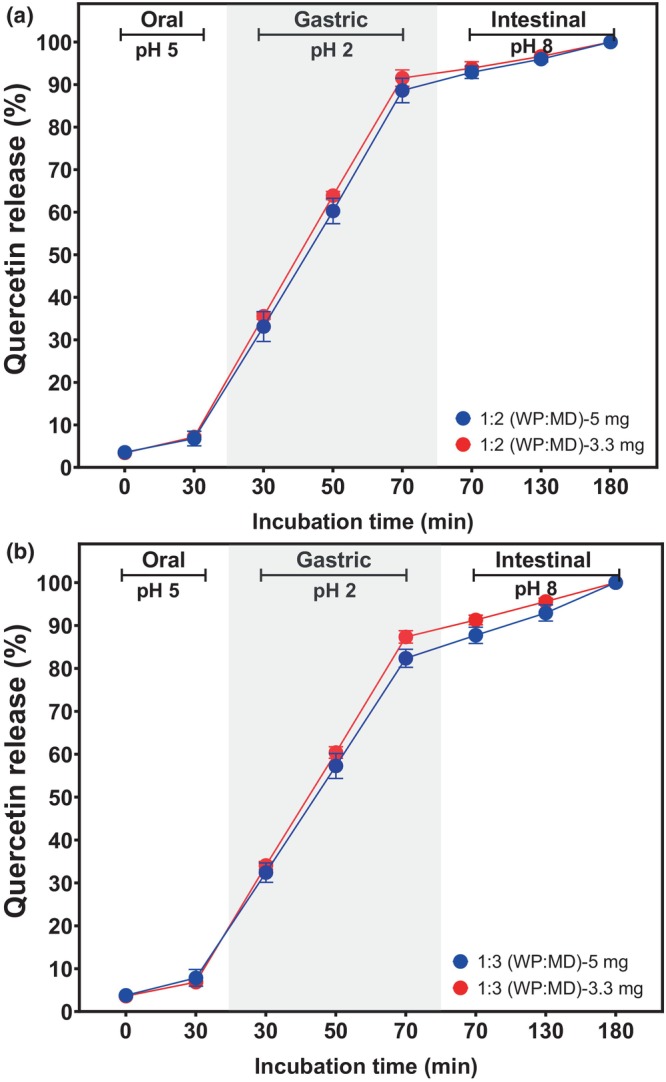
Shows the in vitro gastrointestinal digestion of quercetin‐loaded Maillard conjugates. The mean and standard deviation of two independent experiments are shown. (a) 1:2 ratio (WP:MD); (b) 1:3 ratio (WP:MD); MD, maltodextrin; WP, whey protein. *Statistical difference (*p* < .05) between treatments at the same time and condition.

The dynamics of quercetin release in different Maillard conjugate systems (MCs) were evaluated across oral, gastric, and intestinal stages in an in vitro model (Figure 7). Similar behavior was observed for all MCs along the gastrointestinal tract (GIT). During the oral phase, conditions mimicking chewing and the hydrolytic action of salivary α‐amylase were simulated, maintaining a slightly acidic pH (pH 5) to reflect oral conditions. It was evident that, after the initial 30 min, a modest quercetin release was achieved in all systems, amounting to less than 10% in all cases. The low rate of quercetin release during the oral phase suggests some resistance to early release. Moreover, this may indicate that only a small fraction of quercetin is superficially attached (Ayala‐Fuentes et al., 2023).

On the other hand, altering the pH between the oral and gastric phases significantly influenced quercetin release in all four MC systems. Contrasting the 30‐minute incubation times (Figure 7a,b), it is evident that the transition from pH 5 to pH 2 notably enhanced quercetin release, reaching up to approximately 30%. The release of quercetin increased linearly during the gastric phase (pepsin and acidic conditions, pH 2) with incubation time. This indicates a consistent progression in the decomposition of the Maillard conjugates under the gastric environment as time elapses. Furthermore, this gradual release suggests that the MCs may have the ability to release quercetin in a controlled manner during digestion, resulting in the release of 80%–90% of the encapsulated quercetin after 70 min of incubation. Moreover, treatments loaded with 3.3 mg quercetin/g of MCs showed the highest release rate. Finally, no significant effect of pH change was observed in the intestinal stage. Nevertheless, alkaline conditions (pH 8) and pancreatin activity combined increased quercetin release during the intestinal stage. Accordingly, a quercetin release of 92.3% to 94.4% was achieved after 130 and 180 min of incubation in the intestinal stage.

The data above suggest that MCs could act as vehicles to protect quercetin in the early stages of GIT, while promoting a controlled and sustained release of the drug over time in the gastric and intestinal phases. Therefore, the encapsulation of quercetin by Maillard conjugates may ensure that the drug is adequately delivered for absorption in the intestine. However, in vivo studies are required to validate this behavior.

## 4 CONCLUSIONS

This work aimed to develop Maillard conjugates (MCs) for the encapsulation of quercetin by freeze‐drying, providing a comprehensive understanding of their potential as controlled delivery systems for bioactive compounds. The proportion of whey protein (WP) and maltodextrin (MD) used resulted in Maillard conjugates (blank samples), as evidenced by an increase in the browning index and changes in protein secondary structure (decrease in β‐sheet structures and an increase in α‐helix ratio) found in the blanks (no quercetin loading). The encapsulation method by freeze‐drying showed high encapsulation efficiency (EE), reaching an EE of 87.65% (1:2 WP:MD) and 84.72% (1:3 WP:MD) in treatments loaded with 3.3 mg quercetin/g of MCs. Whereas treatments loaded with 5 mg quercetin/g of MCs obtained a lower encapsulation efficiency of 70.31% and 51.58% when using a 1:2 and 1:3 (WP:MD) ratio, respectively.

The quercetin‐loaded MCs were shown to be spherical microparticles with sizes between 4 to 10 microns and a charge ranging from −9 to −11 mV, suggesting colloidal stability and a size suitable for use as an ingredient for quercetin release in the gastrointestinal tract (GIT). In vitro gastrointestinal release and digestion tests of quercetin demonstrated a complete scenario of the response of MCs under relevant physiological conditions. In that sense, all 4 Maillard conjugate systems exhibited sustained release of quercetin throughout the oral, gastric, and intestinal phases, highlighting the effectiveness of MCs as bioactive delivery systems.

This work not only contributes to the fundamental knowledge of quercetin encapsulation by MCs (using safe and biocompatible materials) but also provides valuable insights for the design of delivery systems for bioactive compounds in food and pharmaceutical applications. Finally, our results open new perspectives for the development of products with improved bioavailability and stability of beneficial compounds in the gastrointestinal tract. Nevertheless, future studies are needed to evaluate the possibility of increasing the drug loading as well as to determine the long‐term stability of the encapsulated quercetin. Moreover, it is necessary to validate the bioavailability and bioaccessibility of quercetin in in vivo models.

## AUTHOR CONTRIBUTIONS

**Angel H. Cabrera‐Ramírez:** Formal analysis (lead); investigation (equal); methodology (lead); validation (lead); visualization (equal); writing – original draft (lead). **Marisol Manríquez‐Medina:** Formal analysis (supporting); methodology (equal); writing – original draft (supporting). **Laura E. Romero‐Robles:** Conceptualization (supporting); formal analysis (supporting); methodology (supporting); validation (supporting); writing – review and editing (supporting). **Rocio A. Chavez‐Santoscoy:** Conceptualization (lead); formal analysis (supporting); funding acquisition (lead); investigation (equal); resources (lead); software (lead); supervision (lead); validation (supporting); writing – original draft (supporting); writing – review and editing (equal).

## FUNDING INFORMATION

This work was supported by the The Tecnologico de Monterrey through the Challenge‐Based Research Funding Program 2023 [E006‐EIC‐GI01‐A‐T23‐D].

## CONFLICT OF INTEREST STATEMENT

The authors declare that the research was conducted in the absence of any commercial or financial relationships that could be construed as a potential conflict of interest.

## ETHICS STATEMENTS

The study did not involve humans or animals as research subjects. No personal data were collected, and no experiments requiring ethics committee approval were performed. Therefore, it was not necessary to obtain informed consent. This study was conducted in accordance with relevant guidelines and regulations.

## Data Availability

Data sharing is not applicable to this article as no new data were created or analyzed in this study.
